# Universal differential equations as a unifying modeling language for neuroscience

**DOI:** 10.3389/fncom.2025.1677930

**Published:** 2025-10-30

**Authors:** Ahmed El-Gazzar, Marcel van Gerven

**Affiliations:** Donders Institute for Brain, Cognition and Behaviour, Radboud University, Nijmegen, Netherlands

**Keywords:** universal differential equations, UDEs, mechanistic models, phenomenological descriptions, neural dynamics, data-driven deep neural networks, DNNs, neural computation

## Abstract

The rapid growth of large-scale neuroscience datasets has spurred diverse modeling strategies, ranging from mechanistic models grounded in biophysics, to phenomenological descriptions of neural dynamics, to data-driven deep neural networks (DNNs). Each approach offers distinct strengths as mechanistic models provide interpretability, phenomenological models capture emergent dynamics, and DNNs excel at predictive accuracy but this also comes with limitations when applied in isolation. Universal differential equations (UDEs) offer a unifying modeling framework that integrates these complementary approaches. By treating differential equations as parameterizable, differentiable objects that can be combined with modern deep learning techniques, UDEs enable hybrid models that balance interpretability with predictive power. We provide a systematic overview of the UDE framework, covering its mathematical foundations, training methodologies, and recent innovations. We argue that UDEs fill a critical gap between mechanistic, phenomenological, and data-driven models in neuroscience, with potential to advance applications in neural computation, neural control, neural decoding, and normative modeling in neuroscience.

## 1 Introduction

As holds for all the natural sciences, modern neuroscience is a scientific discipline whose advancement is fueled by both theoretical and experimental research (Urai et al., 2022; Churchland and Sejnowski, 2016). From a theoretical standpoint, we have witnessed important developments, ranging from detailed mechanistic models of specific neural circuits (Kim et al., 2017; Izhikevich and Edelman, 2008; Felleman and Van Essen, 1991; Bliss and Collingridge, 1993) to grand unified theories of brain function (Van Gelder, 1998; Friston, 2009; Hawkins, 2021; Miller and Cohen, 2001). At the same time, from an experimental standpoint, advances in neurotechnolgy are allowing us to measure (Steinmetz et al., 2021; Urai et al., 2022; Machado et al., 2022) and manipulate (Deisseroth, 2015; Lozano et al., 2019; Blumberger et al., 2018) the activity of many thousands of neurons at an unprecedented scale.

A central challenge in neuroscience is how to integrate these theoretical and empirical insights in order to understand neural mechanisms and develop practical applications. A wide range of modeling approaches have been proposed, each emphasizing different trade-offs between mechanistic interpretability and data-driven flexibility. At one end of the spectrum are white-box or mechanistic models, such as biophysical (Hodgkin and Huxley, 1952; Izhikevich, 2007) and multi-scale (Markram et al., 2015; Breakspear, 2017) simulations of neurons and circuits, which explicitly incorporate known physiology and dynamics. At the other end are black-box or data-driven models, including deep neural networks (Güçlü and Van Gerven, 2015; Yamins and DiCarlo, 2016) and statistical predictors (Yu et al., 2008; Wu et al., 2006; Pandarinath et al., 2018), which excel at capturing variance in high-dimensional data but offer limited interpretability. Between these extremes lie phenomenological (Wilson and Cowan, 1972; Chichilnisky, 2001) and normative (Laughlin, 1981; Todorov and Jordan, 2002; Knill and Pouget, 2004) that abstract away biophysical details to capture computational principles or functional constraints. This diversity reflects both the richness of neuroscience and the limitations of any single paradigm: mechanistic models may be too rigid to account for data variability, while black-box models can miss crucial structure.

Universal differential equations (UDEs) have recently emerged as a promising framework to bridge these approaches (Rackauckas et al., 2020). UDEs extend the dynamical systems perspective that has long guided neuroscience (Favela, 2021), by allowing parts of the governing equations to be learned directly from data while other parts encode prior knowledge. In this way, UDEs combine the interpretability of mechanistic modeling with the adaptability of machine learning. This hybrid formulation is particularly valuable in neuroscience, where experimental measurements are often sparse and noisy, yet rich theoretical knowledge exists across scales, from ion channels to cognitive processes. By embedding flexible function approximators within structured dynamical systems, UDEs enable models that are simultaneously data-adaptive and theory-constrained.

**Figure 1:**
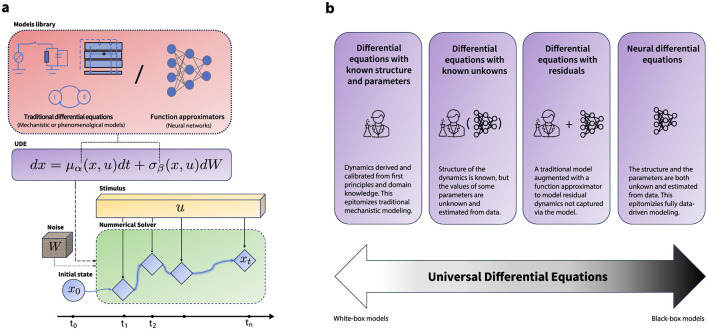
Universal differential equations. **(a)** A schematic illustration of a universal differential equation. The vector field of the differential equation is defined via either an existing model from the literature, or a differentiable universal approximator (e.g. a neural network) or a combination of both. The numerical solver is an SDE-compatible numerical solver, which takes in the initial condition *x*_0_, a geometric Brownian motion generator Δ*W*, the forcing signal *u*, the functions defining the vector fields of the SDE μ and σ, along with their parameters θ. The numerical solver then computes the solution at time *t*. The parameters of the differential equation can then be trained either via automatic differentiation or using adjoints methods. This setup enables the use of a UDE either as universal function approximator on their own or as a part (layer) in a differentiable computational graph. **(b)** The formulation of a UDE encompasses a spectrum of modeling techniques from white-box traditional models to fully data-driven black box models. This flexibility can foster interoperability between different methodological efforts, provide solid theoretical background to face multifaceted challenges in neuroscience modeling across scales and applications, and offer a principled approach to balance between data adaptability and scientific rationale in model development.

Consequently, UDEs are gaining traction across scientific domains where mechanistic models dominate but fall short of fully explaining observations, and where data remain limited (AlQuraishi and Sorger, 2021; Lai et al., 2021; Karniadakis et al., 2021). In neuroscience, this offers a unique opportunity: to unify disparate modeling traditions within a common mathematical framework, situating UDEs as a bridge between white-box and black-box approaches (cf. Figure 1). In doing so, UDEs provide a pathway toward more comprehensive models that integrate across levels of abstraction and link fundamental mechanisms to applied neuroscience (Ramezanian-Panahi et al., 2022).

To motivate UDEs, we begin with a critique on the current landscape of data-driven dynamical systems in neuroscience, highlighting key applications, and challenges, culminating in the motivation for hybrid approaches that combine prior knowledge with empirical data. Next, we delve into the taxonomy of UDEs in the context of stochastic dynamical systems and show how these mathematical objects provide a spectrum of modeling techniques familiar to the neuroscientist spanning from traditional mechanistic white-box models to sophisticated black-box deep learning models. We provide a general recipe for domain-informed training of UDEs for neural system identification and examine the benefits of UDE-based models in emerging applications within the field. We conclude by discussing current challenges and potential future directions. Through this discourse, we argue that UDEs, when augmented with modern machine learning techniques, can serve as the foundational building block for multi-scale modeling in neuroscience, establishing a common language for theory formation and model development.

## 2 Dynamical systems in neuroscience

A prevalent perspective in neuroscience is viewing the brain as a dynamical system, availing the comprehensive toolbox of dynamical systems theory (DST) to the field (Van Gelder, 1998; Izhikevich, 2007; Deco et al., 2008; Breakspear, 2017; Favela, 2021). DST enables the formalization of mechanistic models as systems of differential equations or iterative maps (Hodgkin and Huxley, 1952; FitzHugh, 1961; Izhikevich, 2003) and provides a framework to explain properties of neural systems using intuitive geometrical and topological representations (Deco and Jirsa, 2012; Khona and Fiete, 2022). This view also opens the door to adopt established phenomenological models and tools used in statistical physics to understand neural dynamics (Wilson and Cowan, 1972; Kuramoto, 1975; Buzsaki and Draguhn, 2004). However, mechanistic models often become intractable when scaled to large systems, while phenomenological models may oversimplify biological processes and lack predictive generalizability. For example, van Albada et al. (2015) show that mechanistic models (cortical network simulations) hit limits when scaling, while phenomenological models, such as the Wilson-Cowan equations of population dynamics Wilson and Cowan (1972) or linear-nonlinear models in sensory neuroscience (Chichilnisky, 2001), necessarily simplify neural complexity, thereby limiting their generalizability to high-dimensional neural data.

The unprecedented availability of large-scale datasets in neuroscience has spurred the exploration of data-driven dynamical systems, propelling the field into the era of big data (Landhuis, 2017). These data-driven methods minimize reliance on a-priori assumptions, instead leveraging the rich data available to guide model identification (Brunton and Kutz, 2019). By training these systems to reconstruct empirical observations, they can act as direct surrogates to the system of interest. This attribute makes them especially appealing within neuroscience (Brunton and Beyeler, 2019), a field wherein the systems in question are notoriously complex to model, a unifying theoretical framework is still nascent, and the existing measurement tools do not currently provide a comprehensive representation of the underlying mechanisms. Consequently, data-driven dynamical systems, and specifically deep recurrent neural networks (RNNs) and their variants, are increasingly integrated into a variety of research areas within neuroscience. In systems and computational neuroscience, data-driven dynamical systems are becoming valuable research tools for probing the neural underpinnings of cognitive and behavioral functions (Durstewitz et al., 2023; Vyas et al., 2020; Shenoy et al., 2013; Sussillo, 2014; Barak, 2017; Schaeffer et al., 2022; Mante et al., 2013). In neural control engineering, they are used to develop optimal neurostimulation profiles for treating clinical conditions (Tang and Bassett, 2018; Acharya et al., 2022; Yang et al., 2018; Bolus et al., 2021; Rueckauer and van Gerven, 2023). Similarly, in neural decoding, they are used for reconstructing natural stimuli from neural recordings (Willett et al., 2023; Metzger et al., 2023; Anumanchipalli et al., 2019; Zhang et al., 2019; Livezey and Glaser, 2021), advancing brain-computer interface technologies. Their applications extend to clinical neuroscience, where they are used for bio-marker discovery of psychiatric disorders, patient stratification, and prognosis (Bystritsky et al., 2012; Roberts et al., 2017a,b; Durstewitz et al., 2021).

The shift toward data-driven methodologies in neuroscience can introduce significant technical challenges. These range from data-centric challenges such as high dimensionality, partial observability, non-linearity, process and measurement noise, non-stationarity, and data scarcity, to modeling hurdles such as uncertainty quantification, non-identifiability, and interpretability issues (Durstewitz et al., 2023). This landscape has resulted in a plethora of specialized technical advancements driven by distinct theoretical and practical frameworks (Brunton and Beyeler, 2019; Hurwitz et al., 2021a; Ramezanian-Panahi et al., 2022). A symptom of this status quo is the prevalent dichotomy between model expressivity and interpretability. As researchers opt for more expressive models to capture the intricacies of neural dynamics, they encounter interpretability challenges. This is further exacerbated by optimization challenges that arise either due to the models (e.g. exploding/vanishing gradients in RNNs) or the behavior of the system (e.g. chaos and non-stationarity), entailing highly technical solutions that further fragments neuroscientific practice. Additionally, while the allure of utilizing unbiased expressive models is initially appealing, in the absence of large-scale curated datasets, eschewing prior knowledge often results in ill-posed problems and implausible solutions as highlighted in recent studies (Kao, 2019; Alber et al., 2019; Su et al., 2017). In practical terms, this means that the models become prone to overfitting on spurious correlations and exhibit high sensitivity to design choices that are peripheral to the main task at hand, ultimately leading to issues in generalization and replication across datasets, tasks, and subjects (Maheswaranathan et al., 2019; Schaeffer et al., 2022; Hurwitz et al., 2021a; Han et al., 2023).

Neural differential equations (NDEs) (Chen et al., 2018; Kidger, 2022) have emerged as a powerful tool of choice to implement data-driven dynamical systems. NDEs represent an emerging family of continuous models that utilize neural networks to parameterize the vector fields of differential equations. This integration marries the expressive power of neural networks with the rigorous theoretical foundations established by decades of research in differential equations and dynamical systems theory. While originally popularized as deep neural network models with continuous depth (Chen et al., 2018), recent advancements have burgeoned into a rich spectrum of continuous-time architectures rooted in dynamical systems theory (Tzen and Raginsky, 2019a; Morrill et al., 2021; Li et al., 2020b; Jia and Benson, 2019; Poli et al., 2019; Kidger et al., 2020). Recently, neural ordinary differential equations are being increasingly adopted in computational and systems neuroscience, showing improved performance compared to current approaches (Kim et al., 2021; Sedler et al., 2022; Geenjaar et al., 2023; Versteeg et al., 2023). While this is a promising sign, their current application have only focused on black-box, explicitly discretized versions that do not capture the broader potential of NDEs as a pathway toward a unified scientific modeling language (Shen et al., 2023; AlQuraishi and Sorger, 2021; Wang et al., 2023). This untapped potential can be realized by conceptualizing differential equations as parameterizable, differentiable mathematical objects amenable to augmentation and training via scalable machine learning techniques. Traditional DEs and NDEs can thus be viewed as special cases at the extreme ends of a spectrum.

## 3 Universal differential equations

### 3.1 Mathematical formulation

A UDE is a mathematical model that extends a traditional differential equation by incorporating free parameters whose values can be learnt from data. By including free parameters, a UDE can act as a universal approximator (Cybenko, 1989; Hornik et al., 1989), meaning that it is able to approximate any dynamical system. In their most general form, UDEs are parameterized differential equations that may contain delays, forcing terms, algebraic constraints and/or depend on multiple independent variables (Rackauckas et al., 2020). In this paper, we focus our attention on parameterized forced stochastic differential equations (SDEs) that are able to capture the dynamics of a countable number of state variables (e.g. membrane potentials or population responses) under the influence of process noise and external perturbations (e.g. sensory input or experimental manipulations).

SDEs extend ordinary differential equations by incorporating stochastic processes, enabling the modeling of dynamical systems subject to uncertainty. The key to this extension is the inclusion of a stochastic term that represents random fluctuations arising from either intrinsic or extrinsic factors. A forced SDE makes explicit how the (multi-dimensional) state *x*(*t*) of a system of interest changes as a function of control inputs *u*(*t*) and (Brownian) process noise *W*(*t*) with *t* the time index. This can be succinctly represented as


(1) $$\begin{array}{l} \text{d}x\left(t\right)=\mu_{\alpha }\left(x\left(t\right),u\left(t\right)\right)\text{d}t+\sigma_{\beta }\left(x\left(t\right),u\left(t\right)\right)\text{d}W\left(t\right) \end{array}$$

where μ and σ are drift and diffusion functions, representing the deterministic and stochastic parts of the time evolution of the system. Both μ and σ are parameterized by θ = (α, β), which are the (learnable) free parameters of the system. Note that the time indices in Equation 1 are typically suppressed from the notation for conciseness.

SDEs offer considerable flexibility for modeling stochastic dynamics. This adaptability largely stems from the diffusion term's configuration and Brownian motion properties (Oksendal, 2013; Särkkä and Solin, 2019). For instance, in cases where σ is a constant matrix or a state-independent function, the noise becomes *additive*, rendering it suitable for modeling extrinsic uncertainties such as external, unobserved interactions. Conversely, when σ is a function of the system's state, the noise becomes *multiplicative*, which varies with the system's state, aptly capturing intrinsic uncertainties, such as uncertainties in drift term parameters. Notably, despite the Brownian noise process capturing uncorrelated Gaussian white noise, its interaction with σ enables modeling of non-Gaussian noise distributions. These nuances provide a comprehensive framework for modeling complex dynamical systems with varying types of uncertainty. It is at the modeler's discretion to define the functional form of μ and σ. Ultimately, this functional form should accurately capture the (uncertain) evolution of the state of the system. This is evaluated by computing the solution to Equation 1, which is a distribution over paths *x*(*t*) within some range *t*∈[0, *T*]. When this functional form is unknown, a feed-forward neural network becomes a conventional choice due to their ability to approximate any function. Appendix B provides details on SDE solvers.

### 3.2 Fitting a UDE to data

The key idea behind efficient and scalable training of UDEs is the incorporation of a numerical solver within a differentiable computational graph (Figure 1a). This setup allows gradient back-propagation through the solution of the differential equation, enabling fitting the UDE parameters to observed data given a cost function. There are two primary strategies for this purpose: (i) *discretize-then-optimize*, which involves storing and gradient-backpropgation through all intermediate steps of the solver, providing exact gradients and (ii) *optimize-then-discretize*, utilizing the (stochastic) adjoint-method to approximate gradients at fixed memory cost. Whereas the former is preferred for large-scale learning tasks, the latter preserves continuous-time fidelity and is preferred in control-theoretical settings when stability or theoretical guarantees are critically important.^1^ Effectively, this setup enables the training of a UDE-based model using standard loss functions similar to those used in discrete deep learning models. Nonetheless, given the stochastic nature of a UDE, maximizing the log-likelihood of observations alone can cause the diffusion function to converge to zero (Li et al., 2020a). To counteract this, alternative training strategies, including adversarial methods (Kidger et al., 2021a) or variational inference (Li et al., 2020a; Tzen and Raginsky, 2019a,b), are employed for stochastic UDEs. We explore in further detail the application of variational inference for UDE-based models in Section 4 and the technical details are provided in Appendix C.

### 3.3 A continuum of models

The UDE formulation naturally encompasses a spectrum of modeling approaches from traditional white-box mechanistic models to contemporary expressive black-box deep learning models (Figure 1b). Several modeling scenarios can thus be phrased as a UDE training problem. Here we provide some examples of these scenarios, where we use a subscript θ to indicate free parameters. What these examples have in common is that they fit parameters θ to capture unmodeled variance, which, as we will argue, are themselves amenable to scientific interrogation.

#### 3.3.1 Differential equations with known unknowns

In this setup, the structure of the system dynamics is known or assumed but the values of some parameters are unknown. Training a UDE thus amounts to estimating these unknown parameters from observations. This approach provides a structured and interpretable yet adaptable approach to modeling, capitalizing on domain knowledge or assumptions about the dynamics. This significantly reduces the model search space, and, if correct, would consequently reduce the amount of training required to approximate the dynamical system (Linial et al., 2021; Djeumou et al., 2023; Abrevaya et al., 2023). Consider the following Ornstein-Uhlenbeck (OU) process used as a mechanistic model of the dynamics of a neuron's membrane potential (Laing and Lord, 2009):


(2) $$\begin{array}{l} dx=a\left(m-x\right)\text{d}t+b\text{d}W \end{array}$$

where *x* denotes the membrane potential and θ = (*a, m, b*) are the free parameters. Here, *a* indicates the rate of potential reversion to the mean, *m* represents the resting membrane potential, and *b* is the magnitude of random fluctuations due to synaptic inputs. Here the OU process provides the structure of the model dynamics, while the values of the parameters θ are estimated by fitting the UDE on empirical observations. Hence, the parameters θ provide data-driven estimates of the model parameters that govern stochastic membrane dynamics.

#### 3.3.2 Differential equations with learnable uncertainty

In this setup, the structure of the deterministic dynamics is known or assumed, with unknown parameters, and a function approximator is used to capture intrinsic and/or extrinsic uncertainty about the model. Consider the modern interpretation of a Wilson-Cowan model (Wilson and Cowan, 1972), used to describe the average firing rates of a group of neurons (Sussillo, 2014). This model can be phrased as a UDE to capture stochastic dynamics not captured by the original model as follows:


(3) $$\begin{array}{l} dx=\frac{1}{\tau }\left(-x+Jr\left(x\right)+Bu\right)\text{d}t+\sigma_{\beta }\left(x,u\right)\text{d}W \end{array}$$

where *x* represents the neurons' synaptic currents and θ = (τ, *J, B*, β) are the free parameters. The function *r* is a saturating nonlinear function and *J* is a matrix that represents the recurrent connections between neurons. The vector *u* represent the external input to the network that enters the system through the matrix *B*, and τ represents the time scale of the network. The function σ is a differentiable function approximator (a neural network) that captures both how the dynamics respond to external unobserved inputs (extrinsic uncertainty) and how the dynamics evolve subject to uncertainty about the model parameters (intrinsic uncertainty). Hence, θ denotes the parameters of the traditional model and the function approximator. These parameters are jointly learned by fitting the UDE on observations. Hence, the parameters θ capture essential properties of population dynamics, such as the time constants τ of neuronal population dynamics and the structural, functional and effective connectivity between neural populations, as captured by *J*. This setup allows leveraging interpretable mechanistic deterministic models while embracing the complex stochastic nature that arise empirically when modeling complex systems from partial or noisy observations.

#### 3.3.3 Differential equation with residual learning

This approach starts from an underlying model but assumes that part of the structure of the deterministic dynamics is unknown, which can be captured via a universal function approximator. This approach, also referred to as residual dynamics learning, enables generalizing powerful mechanistic models to handle dynamics not captured by the model. For instance, consider the original Kuramoto model (Kuramoto, 1975), widely used in neuroscience to study synchronization phenomena in systems of coupled oscillators (e.g. neurons, brain regions). A notable shortcoming of this model is its assumption of oscillator homogeneity, implying uniformity across all neurons or regions. However, biological systems often exhibit significant heterogeneity in terms of cell types, regional characteristics, and unobserved inputs. To accommodate these disparities, the Kuramoto model can be augmented with a function approximator, allowing for a more precise representation of neural oscillations. This can be phrased as a UDE


(4) $$\begin{array}{l} dx=\left(\omega +\frac{K}{N}\overset{N}{\underset{j=1}{\sum }}\sin\left(x_{j}-x\right)+f_{\alpha }\left(x\right)\right)\text{d}t+\Sigma \text{d}W \end{array}$$

where *x* is a vector representing the phase of *N* oscillators and θ = (ω, *K*, Σ, α) the free parameters with ω the natural frequencies, *K* a matrix representing the coupling strength between the oscillators, and Σ representing the magnitude of extrinsic random forces acting on the network. The function *f*, parameterized by α, acts as a dynamic corrective mechanism, adjusting for deterministic discrepancies not accounted for in the original model formulation. Note that in this example, all the parameters θ are assumed to be estimated from data, providing an estimate of both the oscillator model parameters as well as the unmodelled dynamics.

#### 3.3.4 Structured neural differential equations

This setup posits that while the coupling architecture between the states (and inputs) of a system is known, the specific dynamic functions governing these states remain unidentified and can thus be approximated via a neural network. This approach is particularly apt for modeling complex, multi-scale, or networked non-linear dynamical systems. Consider the following graph-coupled nonlinear dynamical system generalizing the Kuramoto model, phrased as a UDE:


(5) $$\begin{array}{l} dx=\left(f_{\alpha }\left(x,u\right)+A\overset{N}{\underset{j=1}{\sum }}g_{\alpha }\left(x_{j},x\right)\right)\text{d}t+\Sigma \text{d}W \end{array}$$

where *x* and *u* represent the states and inputs of the system, respectively, and θ = (α, Σ) are the free parameters. The functions *f* and *g* are function approximators describing the local and interconnected system dynamics, respectively. The matrix *A* denotes the adjacency matrix representing the coupling structure, and Σ represents the magnitude of extrinsic random forces acting on the network. In this particular example, the coupling structure is known or assumed based on a-priori assumptions (e.g. structural/functional connectivity), whereas the local and global dynamics functions are completely learned from observations. In essence, either *f* or *g* could be replaced by traditional models allowing combining data-driven and mechanistic/phenomenological modeling across scales.

#### 3.3.5 Neural differential equations

In this configuration, both the parameters and structure of the system's dynamics are unknown. Consequently, the drift and diffusion vector fields of the UDE are entirely described via neural networks as function approximators. This setup represents the epitome of black-box modeling, as it enables the derivation of models directly from observational data circumventing the need for any assumptions about the system's dynamics. A generic UDE in this case can be written in the form of Equation 1 as


(6) $$\begin{array}{l} dx=\mu_{\alpha }\left(x,u\right)\text{d}t+\sigma_{\beta }\left(x,u\right)\text{d}W \end{array}$$

where μ and σ are neural networks with parameters θ = (α, β). This equation can be viewed as a stochastic, continuous-time generalization of discrete-time deep recurrent neural networks prevalent in contemporary machine learning research (Kidger, 2022; Tzen and Raginsky, 2019a,b; Li et al., 2020a). Hence, the parameterized state equations and their ensuing dynamics can be interrogated using analysis techniques developed by the AI and control theory communities (Güçlü and Van Gerven, 2015; Brunton and Kutz, 2019).

### 3.4 Toward informed stochastic models

Generally, all of the presented UDE configurations fill a spectrum between white-box and black-box models under a unified formulation. As one progresses from white-box models toward black-box models, the reliance on empirical data for model identification increases correspondingly, inversely proportional to the number of presupposed assumptions about the underlying dynamics (the more correct the model, the less data needed, and vice versa). In practice, it should be expected that a certain degree of knowledge or hypothesis about the studied system is available. This knowledge should not be constrained to the structure of the dynamics, but could cover all aspects of the computational model (e.g., dimensionality, information about the stimulus or observation modality, scale of noise, expected dynamics, etc.). UDEs simply serve as a universal tool for evaluating this knowledge, or augmenting them to develop scalable models that can be used in several downstream applications (see Section 5).

Crucially, UDEs conceptualize neural processes as continuous-time stochastic processes. This perspective can bring computational models closer to the complex nature of neural processes. This is imperative when modeling neural dynamics, where stochasticity can be traced from the molecular level, with stochastic behaviors in ion channels and synaptic transmission (Hille, 1978; Sakmann, 2013), to the cellular scale where neurons demonstrate unpredictable firing patterns (Tuckwell, 1988). Importantly, stochasticity is not confined to the micro-scale as it escalates to the level of neural populations, where the effects of noise and randomness are not merely incidental but play a crucial role in the functioning and organization of neural systems (Rolls and Deco, 2010; Faisal et al., 2008). The following section delves into leveraging UDEs to develop fully differentiable, informed, probabilistic models for neural system identification.

## 4 Neural system identification

Let us consider a neural system whose dynamics are the realization of a continuous-time stochastic process {*x*(*t*):0 ≤ *t* ≤ *T*} that is potentially modulated by exogenous input *u*(*t*). In practice, we observe neither *x* nor *u* directly but rather have access to stimuli *v*_*n*_ = *v*(*t*_*n*_) and neurobehavioural recordings *y*_*n*_ = *y*(*t*_*n*_), sampled at discrete timepoints *t*_1_, …, *t*_*N*_ with 0 ≤ *t*_*n*_ ≤ *T*. We also use *v*_1:*N*_ and *y*_1:*N*_ to denote these observations across timepoints. Let τ = (*v*_1_, *y*_1_, …, *v*_*N*_, *y*_*N*_) denote a trajectory of stimuli and responses and assume that we have access to a dataset $D=\left\{\tau^{1},\ldots ,\tau^{K}\right\}$ consisting of *k* such trajectories. The goal of neural system identification is to estimate the neural dynamics *x*(*t*) from data D.

We propose to model the underlying stochastic process *x* as the (weak) solution of a latent UDE, and frame the problem of system identification as a posterior inference problem of the distribution *p*(*x*∣*y, v*), which we tackle via variational inference. Accurate resolution of this problem yields multiple benefits. First, it allows inference of the latent (hidden) states of the system in online and offline settings. Second, it allows reconstructing and predicting the system's behavior under various conditions. Third, it provides an expressive probabilistic modeling framework that quantifies uncertainty and incorporates prior knowledge, facilitating robust hypothesis generation and testing.

**Figure 2:**
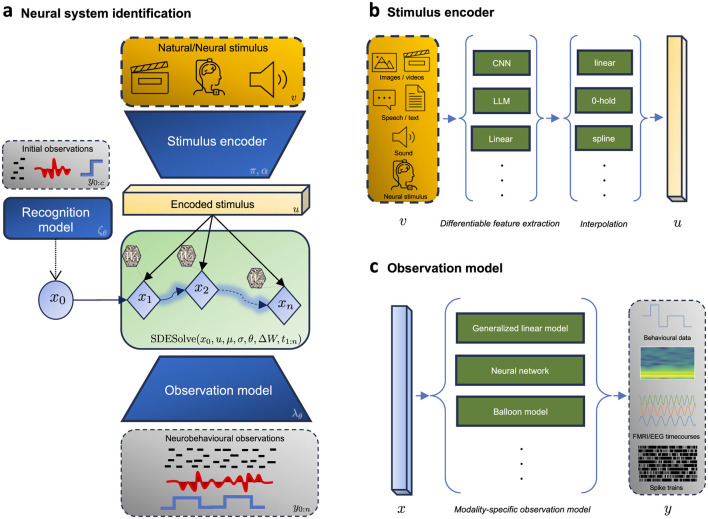
Framework for neural system identification. **(a)** Shows the forward pass (generative mode) during the encoding of a (high-dimensional) stimulus *v* into neurobehavioral observations *y*. This is done through a fully differentiable graph, which consists of (i) a stimulus encoder to encode the stimulus into a lower dimensional continuous representation, (ii) a recognition model to infer the hidden initial state *x*_0_, (iii) a latent dynamics model to model the temporal evolution of the dynamics, and (iv) an observation model to map the latent states into observations. **(b)** Illustrates the formulation of the informed stimulus encoder which is tasked with learning a lower dimensional continuous representation *u* from the discrete (high-dimensional) stimulus signal *v*. **(c)** Illustrates examples of modality-specific observation models to map the latent process into neurobehavioral measurements.

Recent methodological advancements in variational inference for SDE-based models are unlocking new avenues for probabilistic modeling of stochastic dynamical systems (Li et al., 2020a; Tzen and Raginsky, 2019a,b; Ryder et al., 2018; Course and Nair, 2023). This progress can offer transformative potential for neuroscience, specifically for modeling neural systems during complex naturalistic behavior. Here we outline an intuitive recipe, leveraging these techniques in conjunction with neuroscientific domain knowledge, aiming for an informed and data-efficient framework suitable to various applications in the field. As shown in Figure 2, we structure this recipe into four key modules, that is, (i) a stimulus encoder, (ii) a recognition model, (iii) a process model, and (iv) an observation model. This approach was recently adopted by ElGazzar and van Gerven (2024) to estimate the parameters of a stochastic coupled oscillator model which captures the neural dynamics under various motor tasks.

### 4.1 Stimulus encoder

The objective of this module is to map the discrete-time measured stimulus *v* into a (lower-dimensional) continuous-time representation *u* that is suitable for input into the latent dynamics. This is crucial in scenarios where the stimulus is a high-dimensional signal (e.g., images, videos, text, audio), as direct integration into the dynamics model would be computationally expensive. It is also crucial if we wish to sample the latent dynamics at a temporal resolution different from the temporal resolution of the measured input. We formalize this process as


(7) $$\begin{array}{l} u\left(t\right)=\pi {\left\{\alpha_{\theta }\left(v_{\tau }\right)\right\}}_{\tau \leq t} \end{array}$$

where $\alpha_{\theta }:{\mathbb{R}}^{d_{v}}\to {\mathbb{R}}^{d_{u}}$ is a (parameterized) encoding function and $\pi :{\mathbb{R}}^{d_{u}}\times \left[0,T\right]\to {\mathbb{R}}^{d_{u}}$ is the interpolation function that constructs a continuous representation over time. The design of both functions should be guided by the stimulus modality and the context of the scientific question being addressed.

The choice of interpolation scheme π should align with the temporal properties (e.g. smoothness, boundedness, missing data) of the stimulus (Morrill et al., 2022; Lepot et al., 2017), the downstream application (e.g. online vs offline, speed vs accuracy), and the theoretical requirements of the drift term in the dynamics function (controlled differential equation (Kidger et al., 2020) vs forced ODE). For example, while linear interpolation might suffice in most offline scenarios, if the model is to be used in real-time settings (e.g. control) then a rectilinear interpolation scheme is a suitable choice. A general recommended practice is to incorporate time as an additional input channel (Kidger, 2022), especially when the raw input lacks temporal variation, or to model non-autonomous dynamics.

The encoding function α can assume different forms, depending on the research question and data at hand. It could be a simple identity function in case the sensory input is low-dimensional. In case of high-dimensional input, leveraging one or more pre-trained models tailored to specific data modalities could offer a starting point (for instance, a pre-trained convolutional neural network for image data, or a pre-trained language model for text). Alternatively, α may be parameterized by θ and learned directly from data.

Note that α could also be utilized to approximate the posterior distribution *p*(*u*∣*v*) instead of relying on point estimates which would fit well within variational inference framework. However, if the primary interest lies in parameterizing the underlying dynamical system, this added complexity may be unnecessary, as uncertainties about *u* can also be captured through the diffusion term in the UDE. With that said, this approach could be more relevant in downstream applications such as neural decoding (see Section 5).

### 4.2 Recognition model

The objective of this module is to accurately estimate the initial hidden state *x*_0_ of the system. To accomplish this, we define a mapping function that uses a segment of the observed data to infer *x*_0_ as suggested in recent studies (Massaroli et al., 2020; Rubanova et al., 2019). This process involves a backward-running trainable sequential model, denoted as ζ_θ_ [e.g. a RNN or neural controlled differential equation (Kidger et al., 2021a)]. The recognition task can be expressed compactly as


(8) $$\begin{array}{l} x_{0}=\zeta_{\theta }\left(y_{c:0},u_{c:0}\right) \end{array}$$

where *c*∈[0, *N*] denotes the end of the observation interval used for estimating the initial condition. Note that notations *y*_*c*:0_, *u*_*c*:0_ indicate that the intervals are reversed in time. The choice of *c* will depend on the nature of the dynamics or context of the application. For example, in stationary settings, it might suffice to have *c*≪*N*. It is also important to consider, which phenomena is under study. In most cognitive experiments, pre-task/stimulus recordings exist and can be utilized for this purpose. On the other hand, in online settings, dynamically sampling *c* from a pre-defined distribution during training can adapt the model more effectively to real-time variations. In general it is important to ensure that ζ is not overly parameterized to avoid encoding future information about the dynamics as recommended by Massaroli et al. (2020). Additionally, it is worth noting that ζ can be employed to approximate the posterior distribution of the initial state *p*(*x*_0_∣*y*_*c*:0_, *u*_*c*:0_). However, one must consider the added complexity this introduces in the optimization process, especially when variational inference is to be applied across the entire dynamics of the system. Introducing this level of complexity might not always be necessary and could potentially complicate the model without significant benefits in certain contexts.. This approach could be relevant if the underlying dynamics of the system are assumed to be deterministic and autonomous, mirroring many current data-driven dynamical systems in neuroscience (Chen et al., 2018; Sedler et al., 2022).

### 4.3 Process model

The goal of this module is to learn the distribution of the latent stochastic process *x*. This is done by employing a UDE to model the temporal evolution of the initial state *x*_0_, subject to external control *u*, and Brownian motion *W*. This is expressed as before as


(9) $$\begin{array}{l} dx=\mu_{\alpha }\left(x,u\right)\text{d}t+\sigma_{\beta }\left(x,u\right)\text{d}W. \end{array}$$

The design of the UDE should be dependent on domain knowledge about the system in question (Section 3) and the downstream application of the model (Section 5). The parameters of the UDE can be trained along with the rest of the model via variational inference of *x* (Li et al., 2020a; Tzen and Raginsky, 2019b,a) (Appendix C). For the reader familiar with conventional variational autoencoders (VAEs) (Kingma and Welling, 2013), it might be useful to conceptualize this as a variational autoencoder, conditioned on the stimulus (Sohn et al., 2015), with a (learned) expressive prior (Ma et al., 2018), and whose latent space is an SDE-induced continuous stochastic process.

### 4.4 Observation model

Observation models, also known as measurement or emission models, define the probabilistic relationship between the latent states of a system and the observed data. The observation model is formalized as follows:


(10) $$\begin{array}{l} y\left(t\right)=\lambda_{\theta }\left(x\left(t\right),\epsilon \left(t\right)\right) \end{array}$$

where $\lambda_{\theta }:{\mathbb{R}}^{d_{x}}\to {\mathbb{R}}^{d_{y}}$ is the observation function and ϵ(*t*) is observation noise. The above mapping specifies the conditional distribution *p*_θ_(*y*∣*x*) within our probabilistic inference framework. The fidelity of observation models is paramount in the accurate identification of dynamical systems. These models must be tailored to reflect the biophysical constraints in the modality employed, account for specific noise structures, and possibly impose structure if the biological interpetability of latent states is required (Klindt et al., 2017; Seeliger et al., 2021). For instance, point process models can be employed for spike train data (Heeger, 2000). Nonlinear Gaussian models may be apt for local field potentials (LFPs) (Herreras, 2016). Emission models for fMRI should account for the hemodynamic response function (HRF), possibly utilizing convolution or more complex models for regional variation (Friston et al., 2000). Calcium imaging data necessitate nonlinear models to reflect the complex relationship between neural firing and observed signals, with considerations for photobleaching or other imaging artifacts (Rahmati et al., 2016).

## 5 Opportunities in neuroscience

UDEs, when trained for neural system identification, offer the potential to serve as direct substitutes for various (data-driven) dynamical systems currently employed in neuroscience. Here we highlight four emerging applications within the field, outlining both the current challenges and the potential advantages of integrating UDE-based models over existing methodologies.

### 5.1 Explaining cognitive and behavioral functions

A central question in neuroscience is how the brain implements cognitive and behavioral functions. In the past decade, significant progress have been gained by recognizing that these functions arise from coordinated activity within neural populations. This specific population-wide activity appears to be systematically governed by underlying lower-dimensional latent states (Mante et al., 2013; Churchland et al., 2012; Sadtler et al., 2014; Elsayed and Cunningham, 2017). The characterization of latent state dynamics has been a primary focus of many latent variable models (LVMs) (Yu et al., 2008; Cunningham and Yu, 2014; Chen et al., 2018; Glaser et al., 2020; Kim et al., 2021; Hurwitz et al., 2021b; Zhou and Wei, 2020; Schneider et al., 2023). Despite their success in uncovering the neural basis of several phenomena, their utility is contingent upon several factors specific to the neuronal population and experimental settings at hand (Urai et al., 2022; Hurwitz et al., 2021a). These factors include low dimensionality of neural population activity, autonomous latent trajectories, and settings where most of the variance is explained by behavior. Consequently, most breakthroughs in this domain have arisen from studies of highly stereotyped behaviors within simple contexts, particularly in neural populations in the motor cortex. Additionally, several open challenges remain for current LVM approaches (Vyas et al., 2020), such as delineating the specific input-output structures of brain regions, understanding inter-neuronal population influences, modulating of local and global dynamics, and integrating both anatomical and functional constraints.

We posit that UDEs provide principled solutions to address these issues, making them a suitable candidate for multi-scale modeling of neural dynamics during complex behaviors. Note that LVMs, by design, will mix all sources of neural variability in the latent space, obscuring the interpretation of the latent dynamical system, especially for complex behaviors or diverse stimulus ensembles. UDEs offer a solution by imposing structured dynamics in the latent space, which can additionally improve model expressiveness and reduce the search space. The rich history of differential equations in neuroscience provides a foundation for such structure, which can be utilized to construct the drift term of a UDE and leverage the variational inference framework to infer their parameters. The effectiveness of this approach also crucially depends on the construction of appropriate encoder and observation models since this will affect the nature of inferred latent dynamics.

**Figure 3:**
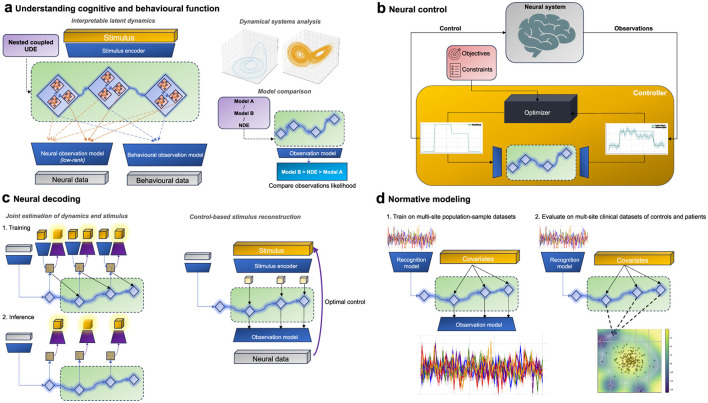
UDE-based models across different applications in neuroscience **(a)** UDEs can be used to represent the underlying latent dynamical system to understand how neural dynamics give rise to computations and ultimately behavior. This could be achieved via imposing multi-scale dynamics models, comparing different dynamics models, and dynamical systems analysis. **(b)** UDEs can be used for model-based closed loop control of neural systems via model predictive control methods. **(c)** UDE models trained for neural encoding can also be leveraged for neural decoding of the stimulus from neural observations. This could be achieved via explicitly training UDEs to jointly estimate latent dynamics and the stimulus in a supervised fashion or via using control-theoretic methods to infer the applied stimulus from observational data. **(d)** UDE models can be employed for capturing population-average neural dynamics utilized for patients stratification in a normative modeling framework. First UDEs can be trained on population-level data, and then used as a scoring function to detect outliers at inference time.

A particularly intriguing possibility that arises from the hybridization of mechanistic differential equations and neural networks in UDEs is developing expressive tractable multi-scale models. By imposing a-priori dynamics (with trainable parameters) at one scale while learning complex dynamics at another, we can combine different level of descriptions within a unified framework (Figure 3a). For example, existing single-neuron differential equations could model micro-scale dynamics, while neural networks could capture abstract inter-population macroscopic dynamics. Conversely, neural networks might model abstract microscopic dynamics within brain regions, and phenomenological models, guided by structural or functional insights, could represent macroscopic dynamics between brain regions. In this setup, UDEs function as a system of nested, coupled differential equations (Koch et al., 2023). Again the ability to assign such correspondences would require the design of (low-rank) observation models that allow for the allocation of specific latent states to particular physical observations.

Another dimension of interpretability, which does not hinge on interpretable latent states, emerges from applying dynamical systems theory. By examining the vector field of the trained UDEs to identify dynamical phenomena and structures of interest (such as attractors, limit cycles, bifurcations, etc.), we can generate insights into how the dynamical system facilitates computations underlying cognition and behavior. This approach, drawing on principles from dynamical systems theory, has gained popularity in the analysis of RNN-based models (Sussillo and Barak, 2013; Durstewitz et al., 2023; Vyas et al., 2020).

Contemporary methodologies for addressing stochasticity in neural dynamics often resort to simplified models of noise. Common approaches include using probabilistic initial states coupled with deterministic dynamics, or incorporating dynamics perturbed by additive Gaussian or Poisson noise, as well as employing hidden Markov models (Linderman et al., 2017; Laing and Lord, 2009; Pandarinath et al., 2018). While these methods can be adequate for modeling neural activity in autonomous tasks, discrete decision-making processes, or certain brain regions, they frequently fall short in encapsulating the full complexity inherent in higher-order brain functions or intricate decision-making scenarios. In such contexts, noise plays a more substantive role than just a source of randomness; it becomes a fundamental component of neural coding and behavior formation (Rolls and Deco, 2010; Faisal et al., 2008). UDEs represent a significant leap in the ability to model arbitrarily complex noise distributions. This is achieved through the integration of a high-capacity function approximator such as a neural network conditioned on the state in the diffusion term. By appropriately choosing the function σ(*x, u*), which encodes a state- and control-dependent diffusion matrix, the diffusion term can capture a wide range of noise phenomena. For example, constant or state-dependent σ produces additive or multiplicative Gaussian fluctuations, diffusion approximations σ(*x, u*)d*W* can approximate non-Gaussian variability, such as Poisson-like spike count fluctuations, while multiple or correlated Wiener processes encoded in the off-diagonal terms of the diffusion matrix allow structured or network-level noise. Finally, it is important to note that UDEs are not bound to conditioning on Brownian noise since SDEs can also be driven by jump processes or more general Lévy processes (Øksendal, 2003; Gardiner, 2009; Faugeras and Inglis, 2015).

UDEs offer a compelling framework for model comparison in neuroscience. Essentially, by leveraging the probabilistic inference framework, we can configure the prior UDE to reflect various theoretical models about how neural processes unfold over time. The crux of this approach lies in determining whether UDEs, when structured to reflect specific hypotheses, can improve the log-likelihood of observed data over models with non-specific or generic priors under identical training data conditions. Such a comparison is not hypothesis testing in the formal Bayesian sense, but rather a way to evaluate the relative effectiveness of different (potentially highly expressive) dynamical models in explaining neural data, which otherwise would not be tractable in standard Bayesian settings (Grimmer, 2011).

Beyond hypothesis testing, one of the most compelling aspects about adopting UDEs for neuroscience is the potential for automated scientific discovery. This process is typically enabled by using sparsity-promoting optimization techniques (Brunton et al., 2016; Schmidt and Lipson, 2009) to recover compact closed-form equations from a large database of basis functions. Within the framework of UDEs, this is viewed as a post-hoc step involving symbolic distillation of the function approximators to recover missing terms and auto-correct existing mechanistic models (Cranmer et al., 2020; Rackauckas et al., 2020). Unlike several scientific disciplines which are starting to embrace this approach (Raissi, 2018; Keith et al., 2021; Davies et al., 2021; Duraisamy et al., 2019; Kusner et al., 2017; Choudhary et al., 2022), this remains an underexplored opportunity in neuroscience with a potential to generate data-driven hypotheses in the form of interpretable algebraic expressions (Wang et al., 2023).

### 5.2 Neural control

The confluence of neuroscience and control theory is becoming increasingly pronounced, spurred by the potential of brain-computer interfaces (BCI) and neurostimulation for clinical interventions, sensorimotor augmentation, and functional brain mapping (Yang et al., 2018; Acharya et al., 2022). This burgeoning field, also termed “neural control engineering” (Schiff, 2011), is predicated on the notion that the brain is fundamentally a complex, adaptive system, amenable to modeling and control using established engineering and control theory principles. In practice, the predominant focus has been on open-loop control methods for neural systems. However, there is a growing consensus that transitioning to a closed-loop control paradigm is imperative to improve reliability, safety and energy efficiency (Ramirez-Zamora et al., 2018; Särkkä and Solin, 2019). Particularly, model-based closed-loop control aids safety via allowing the development and validation of control strategies in-silico (Rueckauer and van Gerven, 2023). It also promotes interpretability by facilitating causal analysis of the models (Imbens and Rubin, 2015). Additionally, it paves the way for integrating the latest advancements at the intersection of control theory and machine learning in a data-efficient manner (Moerland et al., 2023).

However, unlike the typical engineering context in which model-based control methods are being developed, the brain posits a number of additional challenges (Schiff, 2011). These challenges encompass its high dimensionality, the multitude of constituent subsystems, limited data availability, inherent stochasticity, the myriad of spatio-temporal scales influencing system behavior, and technological constraints in sensing and actuation, among others. Consequently, there is a pressing need for tailored model-based frameworks specific to the brain (Ramirez-Zamora et al., 2018). UDE-based models provide principled solutions to address these challenges, rendering them a strong candidate to develop models for neural control (Figure 3b). The integration of mechanistic models with data-driven models in UDEs offer a way to navigate the current dichotomy of expressiveness versus data requirements. Fully mechanistic models, or linear data-driven models, while being less reliant on extensive data and potentially more interpretable, may compromise on prediction accuracy. Conversely, expressive non-linear data-driven dynamical systems can provide better prediction fidelity but necessitate substantial, often personalized, supervised datasets—a requirement that can be challenging in practice.

One of the main concerns of closed-loop control system is its robustness to external disturbances and uncertainties. These uncertainties as discussed can come from all kind of sources in the neural system, ranging from epistemic uncertainty about the system to aleatoric uncertainty as generated by sensor, process and actuator noise. Such uncertainties is critical for safe and reliable design of control policies. A latent UDE model can provide a principled approach to estimate and disentangle these uncertainties even for highly expressive models in a tractable efficient manner. Latent UDEs facilitate the use of both linear and nonlinear control methods. Through the notion of amortized priors, a distilled, simpler (possibly linear) model can be approximated from a complex dynamics model for real-time application, enabling optimal linear control strategies. In applications where linear models are sub-optimal, we can still use an amortized non-linear UDE for system identification. The availability of a fully differentiable dynamics model then unlocks advanced control strategies, such as model-based reinforcement learning and (gradient-based) model predictive control (Moerland et al., 2023; Djeumou et al., 2023).

For real-time applications, striking a balance between prediction accuracy and computational efficiency is paramount. UDEs, being compatible with adaptive numerical solvers, can thus be tailored to offer this trade-off crucial during real-time application via the choice of the numerical solver, or the trade-off between error tolerances and speed (Yildiz et al., 2021). Additionally, their continuous-time nature allows for handling irregularly sampled heterogeneous data and guarantees adaptive continuous control in the absence of observations, ensuring safety of operation (Lewis et al., 2012).

### 5.3 Neural decoding

Neural decoding utilizes activity recorded from the brain to make predictions about stimuli in the outside world (Rieke et al., 1999; Horikawa et al., 2013; Anumanchipalli et al., 2019; Seeliger et al., 2018; Metzger et al., 2023). Such predictions can serve various purposes, from enabling communication interfaces and controlling robotic devices to improving our understanding of how brain regions interact with natural stimuli. As a result, neural decoding is rapidly becoming an indispensable framework in neuroscience (Donoghue, 2002; Zhang et al., 2019). Current approaches can be roughly broken down into two categories, in which the decoding algorithm is based on either (deep learning-based) regression techniques (Warland et al., 1997; Horikawa et al., 2013; Seeliger et al., 2018; Anumanchipalli et al., 2019; Metzger et al., 2023) or Bayesian methods (Pillow et al., 2011).

The architecture introduced earlier for system identification in Section 4 can be viewed as a neural encoding model. One option to reconfigure the architecture for neural decoding is to simply invert the input and output (and their corresponding encoder/decoder networks) during the training process to obtain a feasible trainable akin to modern supervised decoding models. A more powerful alternative is to utilize the same model for encoding, to also do decoding (Paninski et al., 2007; Kriegeskorte and Douglas, 2019). Despite the ill-posed nature of the problem, we propose two approaches commonly used in modern control literature, utilizing the same architecture introduced earlier to potentially approach this problem in a tractable manner.

The first approach includes a modification to the architecture introduced earlier in Section 4 to extend the probabilistic inference framework to approximate the posterior distribution *p*(*v*∣*y*). This involves updating the stimulus encoder to generate the parameters of an approximate posterior instead, a tractable prior over *p*(*u*), and an additional decoder head to output the reconstructed stimulus (Figure 3c). We can then update optimization function accordingly. The second approach is to frame the problem of stimulus inference as a separate optimization problem akin to optimization problems solved in control applications.

Recently, Schimel et al. (2021) adopted the latter idea, by utilizing an iterative linear quadratic regulator (ILQR) within the recognition model of a sequential VAE. This method is used to estimate the initial state and infer any unobserved external inputs driving the system, demonstrating success on both synthetic and real-world neuroscience datasets. However, they note that the approach can be prone to local minima and may struggle with significant mismatches between the employed prior over the input and the posterior. They suggest that independently modeling process noise could mitigate these issues. This is an inherent advantage of UDEs, which naturally incorporate independent modeling of process noise, and could utilize this control-based approach for decoding.

### 5.4 Normative modeling

Normative modeling is an increasingly popular framework in clinical and developmental neuroscience that aims to characterize the *normal* variation in brain features across a population and then assess individual deviations from this norm (Marquand et al., 2016; Insel et al., 2010; Bethlehem et al., 2022). This approach offers a statistical framework to correlate individual differences in brain metrics such as connectivity patterns, structural attributes, or task-induced responses with behavioral or clinical indicators. The appeal of normative modeling is becoming particularly pronounced in psychiatry. As the discipline increasingly recognizes the heterogeneity inherent in these measures (Kapur et al., 2012), there is a concerted move toward eschewing symptom-based labels in favor of biologically grounded metrics. So far the emphasis in normative modeling has been on behavioral, structural neuroimaging, or static summaries of functional data (Wolfers et al., 2020; Zabihi et al., 2019; Rutherford et al., 2023). Developing normative models for dynamic representations of functional neuroimaging (e.g. EEG/fMRI timecourses) data remains a formidable challenge but necessary to characterize the majority of psychiatric disorders (Marquand et al., 2019; Brodersen et al., 2014; Rutherford et al., 2022; Gazzar et al., 2022). The challenge lies in capturing the high dimensional spatio-temporal dynamics of brain activity, which is further complicated by factors such as inter-subject variability, measurement noise (including physiological and scanner-related noise), and often limited sample sizes.

UDE-based models can provide a solution to navigate these challenges. Their flexible structure is adept at encapsulating a wide range of variability through the a complex diffusion term while capturing the population average behavior in the drift term. The drift component of the UDE can further be parameterized with fixed arguments reflecting observed covariates within the population such as age, sex, scanning site, behavioral metrics, etc. At training time, the model can be optimized to reconstruct the neural data from observed variations and initial observations of population-sample or control only multi-site datasets (Rutherford et al., 2022). At test time, stratification is done via running the model on both control and patients and comparing their latent dynamics (or observations) log-likelihood (Figure 3d). This approach further provides a principled interpretable method to understand psychiatric disorders through the lens of network dynamics (Durstewitz et al., 2021; Anyaeji et al., 2021; Segal et al., 2023).

## 6 Outlook and challenges

There is a growing consensus that solutions to complex science and engineering problems require novel methodologies that are able to integrate traditional mechanistic modeling approaches and domain expertise with state-of-the-art machine learning and optimization techniques (Raissi et al., 2019; Alber et al., 2019; Willard et al., 2022; Cuomo et al., 2022; AlQuraishi and Sorger, 2021; De Bézenac et al., 2019). In this vein, we explore the potential of universal differential equations (Rackauckas et al., 2020) as a framework to facilitate this integration in neuroscience. This endeavor is centered around the motivation of establishing a common potent language for modeling across the field.

In the realm of machine learning, differential equations and neural networks are increasingly being recognized as two sides of the same coin through the concept of neural differential equations (Kidger, 2022). For example, a residual neural network can be viewed as a discretized variant of a continuous-depth ODE with a neural network parametrizing its vector field (Chen et al., 2018). Similarly, an RNN is equivalent to a neural controlled differential equation or a forced neural ODE, discretized via Euler approximation (Kidger et al., 2020). A convolutional neural network is roughly equivalent to the discretization of a parabolic PDE (Li et al., 2020b,c). At first glance, these insights might not be entirely novel or exciting for a field such as computational neuroscience, which has been successfully applying RNNs and their variants as discretizations of ODE models for over a decade. What is exciting however, is that this offers a fresh perspective to view and connect models from different scales of organization and levels of abstraction in neuroscience under one potent framework.

Beyond the advantages discussed throughout the rest of the paper, this perspective brings us closer to the language of dynamical system theory and classical differential equation literature. This alignment provides principled solutions to optimization and interpretation challenges in existing RNN-based models. For example, rough differential equations and log-ODEs might offer improved handling of long time series data (Morrill et al., 2021), partial differential equations (PDEs) create a natural bridge between dynamical systems and spatial domains (Li et al., 2020b), while SDEs offer structured ways to handle uncertainty (Laing and Lord, 2009). Being inherently continuous, these models adeptly handle irregularly sampled data and are compatible with powerful adaptive numerical solvers. All of these solutions could be explored and parsed through the formalism of UDEs.

Our focus in this paper is primarily on stochastic variants of UDEs, underscoring the empirical challenges in modeling neural systems. Despite advancements in neural recording technologies, the data obtained represents only a small, noisy subset of underlying mechanisms. Recent hypotheses suggest that behaviorally relevant neural dynamics may be confined to lower-dimensional spaces (Mante et al., 2013; Churchland et al., 2012; Sadtler et al., 2014; Elsayed and Cunningham, 2017). Yet, these representations may not consistently translate across time, task contexts, and brain regions. Challenges such as non-stationarity, intrinsic stochasticity of neural mechanisms, and the absence of a robust theoretical modeling framework raise doubts about our capacity to accurately model neural systems. Consequently, current models may over-rely on conditions where dynamics are autonomous or predominantly behavior-driven, as observed in cognitive experiments involving stereotyped tasks. These models are less effective in complex scenarios where the multi-scale spatial and temporal aspects of neural dynamics, such as in naturalistic behavior, become prominent. UDEs, as a form of SDEs, provide a framework to acknowledge and address these uncertainties by modeling neural processes as stochastic phenomena. Leveraging high-capacity function approximators in conjunction with SDE theory offers a pathway to navigate this challenging terrain.

We have presented a recipe for informed training of UDE-based models for neural system identification. This recipe leverages recent advancements in stochastic variational inference for SDE-based models and can be easily tailored to different downstream applications. While similar strategies are showing promising results across different applications (Course and Nair, 2023; Djeumou et al., 2023; Fagin et al., 2023), practical implementation in neuroscience is still warranted. Additionally, there remains several open practical questions and simplifying assumptions that warrant further research.

One assumption is modeling all uncertainties as standard Brownian motion within our dynamical systems. This perspective, while potent and aligning with the central limit theorem, can oversimplify real-world scenarios, where noise characteristics differ in bias and time-dependence. Recent advances in learning neural SDEs for fractional Brownian motion types offer avenues to better represent these complexities (Tong et al., 2022; Daems et al., 2023).

Secondly, the variational inference approach models the probability distributions of latent states and observations but makes point estimates for other inferred values, like initial states, model parameters, and encoded stimuli. While from a pragmatic point of view, this can be warranted, especially given the nature of SDE-based dynamical systems, these practices could significantly influence the final model, and prevent formal Bayesian model comparison. With that said, recent developments in this area are promising and rapidly evolving. For example, Course and Nair (2023) provides an approach to jointly estimate the probability distribution of the SDE parameters using a reparametrization trick for Gaussian-Markov processes. This could be beneficial in our setup if the drift in the UDE is dependent on the parameters of the mechanistic model alone and no neural networks are used. Alternatively, Zeng et al. (2023) provides an efficient approach for probabilistic inference of latent neural SDEs that operate on homogeneous manifolds, an assumption ubiquitous in neuroscience. These developments pave the way toward fully probabilistic treatment of our models.

Lastly, the field of neural differential equations is relatively nascent compared to established deep learning practice. Challenges remain, particularly in gradient back-propagation through numerical solvers. In some scenarios where the dynamics are stiff or discontinuous, training via automatic differentiation with high-order adaptive numerical solvers can be very expensive in terms of memory and speed (Ma et al., 2021). Alternatively, training via adjoint-sensitivity methods can be more memory-efficient but still remains slow and results in biased gradients. Innovative solutions like algebraically reversible solvers (Kidger et al., 2021b), and stochastic automatic differentiation (Arya et al., 2022) are emerging, but their mainstream adoption is still in early stages.

With that said, the challenges discussed are not unique to neuroscience but resonate across various scientific disciplines. This burgeoning field of scientific machine learning is a collaborative and innovative arena, marked by rapid advancements. The recent surge in open-source software and packages (Rackauckas and Nie, 2017; Rackauckas et al., 2019; Lienen and Günnemann, 2022; Drgona et al., 2023; Kidger and Garcia, 2021) centered around neural and universal differential equations specifically, and automated model discovery in general, underscores the growing interest and recognition of a new era of scientific discovery (Wang et al., 2023). Neuroscience is poised to embrace this new era, to push the boundaries of our current understanding of the brain and advance practical applications in the field.

## Footnotes

^1^Other methods, such as reversible solvers or forward sensitivity methods, also exist but are less common. For a detailed comparison of NDE automatic differentiation techniques, refer to Ma et al. (2021) and Kidger (2022).
