# Supplementary material for: Universal differential equations as a unifying modeling language for neuroscience
Source: Front Comput Neurosci. 2025 Oct 30;19:1677930. doi: 10.3389/fncom.2025.1677930 (PMC12611869; doi:10.3389/fncom.2025.1677930)
Supplement: Supplementary file 1 [file Data_Sheet_1.pdf]

# Supplementary Material for Universal Differential Equations as a Unifying Modeling Language for Neuroscience

**Ahmed El-Gazzar[1,*] and Marcel van Gerven[1]**

[1]Donders Institute for Brain, Cognition and Behaviour, Radboud University, Nijmegen, the Netherlands
[*]e-mail: ahmed.elgazzar@donders.ru.nl

## ABSTRACT

This document contains supplementary information supporting the main text.

## A Glossary

**adjoint-method** A numerical method for efficiently computing the gradient of a function or operator in a numerical optimization problem. In the context of neural differential equation it used for memory-efficient gradient back-propagation through the solution. 1

**adversarial methods** Techniques in machine learning, particularly in neural networks, where models are trained against adversaries to improve robustness and performance, often used in generative models and for enhancing model security. 1

**aleatoric uncertainty** Pertains to uncertainty inherent to a system due to variability or randomness that cannot be reduced with more information. This type of uncertainty is intrinsic to the process or environment being observed and is often represented probabilistically. 1

**amortized priors** In the context of machine learning and statistics, amortized priors refer to the concept where (some of) the parameters of the prior distribution are estimated across multiple observations or data points, rather than being entirely pre-defined. This approach allows for more efficient computation and learning, as the prior information is 'amortized' or spread out over the entire dataset, enabling the model to leverage shared information across different data instances. 1

**biophysical models** Models grounded in physiology and biophysics, with equations directly encoding known mechanisms such as ion channel kinetics, membrane dynamics, and synaptic transmission. 1

**Brownian motion** A type of stochastic process that models random motion, often used to represent particle movement. Brownian motion is a central concept in the theory of stochastic processes and is characterized by continuous, nowhere differentiable paths. 1

**deep neural networks** A class of machine learning models based on artificial neural networks with multiple layers, capable of automatically learning hierarchical representations of data. 1

**epistemic uncertainty** Refers to uncertainty stemming from a lack of knowledge or information about a system or environment. It is often associated with the limited data or incomplete understanding of the underlying mechanisms of the system. Epistemic uncertainty can, in principle, be reduced through additional data collection, research, or analysis. 1

**gradient back-propagation** A fundamental algorithm in neural network training, gradient back-propagation computes the gradient of the loss function with respect to the weights of the network. This process involves a forward pass to calculate outputs and loss, followed by a backward pass to calculate gradients. These gradients are then used to adjust the weights, optimizing the network's performance. It is crucial for training deep neural networks, enabling the effective learning of complex patterns in data . 1

**Itô calculus** A branch of mathematical analysis that deals with stochastic integration and differentiation. Itô calculus is fundamental in the study of stochastic differential equations and is widely used in various fields such as finance and physics. 1, 3

**Lipschitz-continuous and bounded** A property of functions where there exists a constant such that the absolute difference in function values is bounded by this constant multiplied by the absolute difference in input values. This property ensures stability and well-behavedness in mathematical models. 1

**manifold hypothesis** A foundational concept in the field of machine learning, suggesting that high-dimensional data (such as images, text, or neural data) usually lie on lower-dimensional manifolds within the high-dimensional space. This implies that although the data might exist in a space with a large number of dimensions, the intrinsic dimensions that actually govern the data's structure are much fewer. This lower-dimensional subspace or manifold captures the essential features and relationships in the data, making it possible to simplify and understand complex datasets. 1

**multi-scale models** Models that integrate mechanistic and data-driven components across biological scales (molecular $\rightarrow$ neuronal $\rightarrow$ systems $\rightarrow$ behavior). 1

**normative models** Models based on optimality principles or functional constraints, such as efficient coding and reinforcement learning. 1

**numerical solver** An algorithm that approximates solutions to mathematical problems, especially differential equations, where analytical solutions are not feasible. They employ iterative numerical methods to simulate complex systems, crucial in various scientific and engineering applications. Their accuracy and methodology are adapted to suit different equation types and computational needs . 1

**ordinary differential equations** An ordinary differential equation (ODE) is an equation which consists of one or more functions of one independent variable along with their derivatives. A differential equation is an equation that contains a function with one or more derivatives. But in the case ODE, the word ordinary is used for derivative of the functions for the single independent variable. ODEs are used to model the rate of change of the state of a system and have applications across various scientific disciplines, including physics, biology, and engineering. 1

**path** In the context of stochastic processes, a path refers to a realization of the process, representing a possible sequence of states or events that the process can take over time. 1

**phenomenological models** Simplified models of observed neural dynamics that abstract away detailed mechanisms while reproducing characteristic behaviors. 1

**statistical predictors** Models that infer relationships between input variables and target outcomes using explicit statistical assumptions. 1

**stochastic processes** Processes that involve a sequence of random variables changing over time. Stochastic processes are used to model systems or phenomena that are inherently unpredictable and have applications in fields like finance, physics, and biology. 1

**Stratonovich calculus** A formulation of stochastic calculus, similar to Itô calculus, but uses a different definition of the stochastic integral. The Stratonovich integral is often used when the stochastic system is derived from a physical model. 1, 3

**universal approximator** A universal approximator is a parameterized object capable of representing any possible function in some parameter size limit. Common universal approximators in low dimensions include Fourier or Chebyshev expansions, while common universal approximators in high dimensions include neural networks. 1

**variational inference** A method in Bayesian inference for approximating probability densities. This technique is widely used in machine learning for fitting complex models to data, especially in scenarios where exact inference is computationally infeasible. 1

## B Solving stochastic differential equations

Consider the general form of an SDE

$$\mathrm{d}x = \mu(x,t)\mathrm{d}t + \sigma(x,t)\mathrm{d}W \tag{1}$$

where $\mu$ and $\sigma$ represent the deterministic drift and stochastic diffusion components, respectively. This non-autonomous (time-varying) formulation also includes forced SDEs since we may use the substitutions $\mu(x(t),t) = \mu'(x(t),u(t))$ and $\sigma(x(t),t) = \sigma'(x(t),u(t))$. The system's state $x(t)$ in this case is a stochastic process, and can be obtained by solving the differential equation as follows

$$x(t) = x(0) + \int_0^t \mu(x(s),s)\mathrm{d}s + \int_0^t \sigma(x(s),s)\mathrm{d}W(s)\,. \tag{2}$$

Two main challenges arise in attempting to solve this equation. Firstly, Brownian motion is almost surely nowhere differentiable, rendering standard (Riemann) integration infeasible for the last term. This necessitates the application of stochastic calculus with one of two primary frameworks: Itô calculus, or Stratonovich calculus. While each has its typical applications and advantages, they coincide when $\sigma$ is a fixed matrix, and the choice becomes arbitrary when it is a learned function. Secondly, deriving analytical solutions for non-trivial SDEs is typically infeasible. Thus we resort to numerical solvers compatible with SDEs[1] (e.g. Euler–Maruyama, Milstein or stochastic Runge–Kutta integration) to approximate the solution at discrete time points via

$$x_{n+1} \approx \texttt{SDESolve}(x_n, \mu, \sigma, \Delta W, \Delta t) \tag{3}$$

for $0 \leq t_n \leq T$ with $\Delta W$ a Brownian increment and $\Delta t = t_{n+1} - t_n$ the time step.

SDEs have seen widespread application for modeling real-world phenomena, such as asset prices in finance[2,3], particle dynamics in physics[4], population dynamics in ecology[5], and allele expression in genetics[6]. Historically, however, constructing the structure of SDEs has relied heavily on substantial domain expertise. Additionally, fitting the parameters of SDEs to data has been dependent on techniques that fail to scale favorably[7–9]. These limitations have often resulted in oversimplified models lacking the representational capacity to accurately capture the intricacies of complex processes. The recent advent of universal differential equations heralds a new era of opportunities to develop expressive and scalable SDE models for modelling and estimating complex dynamical systems.

## C Variational inference of latent UDE models

To perform variational inference in the context of latent continuous stochastic processes, we need to define a reasonable and tractable family of path distributions for both the prior and the approximate posterior. Following the approach of[10], we may employ two SDEs to represent these distributions. Specifically, here we can define our (amortized) prior as a UDE and our approximate posterior another black-box UDE. Note that the term prior here refers to our main generative UDE, and the approximate posterior is an auxiliary UDE that is used only during training. The UDEs can be written as:

$$\begin{aligned} \mathrm{d}x &= \mu_\theta(x,u)\mathrm{d}t + \sigma_\theta(x,u)\mathrm{d}W\,, & x(0) &= x_0 & &\text{(prior)} \\ \mathrm{d}\tilde{x} &= \mu_\phi(\tilde{x},y,u)\mathrm{d}t + \sigma_\theta(\tilde{x},u)\mathrm{d}W\,, & \tilde{x}(0) &= x_0 & &\text{(approximate posterior)} \end{aligned} \tag{4}$$

where $\phi$ are the variational parameters. Note that both processes share the same diffusion $\sigma_\theta$, a decision which guarantees that the Kullback-Leibler (KL) divergence between the two probability measures they induce is finite (under some mild conditions)[10]. This KL divergence can be defined using Girsanov's theorem[11] as

$$\mathrm{KL}(Q||P) = \mathbb{E}_Q\left[\int_0^T \left(\frac{1}{2}\left\|\sigma_\theta(\tilde{x},u)^{-1}\left(\mu_\phi(\tilde{x},y,u) - \mu_\theta(\tilde{x},u)\right)\right\|^2\right)\mathrm{d}t\right] \tag{5}$$

where $Q$ and $P$ denote the path space probability measures induced by the approximate posterior and prior UDEs, respectively. Intuitively, this KL divergence resembles the integrated difference over the time horizon $[0,T]$ between the prior drift $\mu_\theta$ and posterior drift $\mu_\phi$, scaled by the diffusion $\sigma_\theta$. This divergence can be estimated up to a constant with Monte Carlo, sampling trajectories from the dynamics given by the approximate posterior.

The neural system identification framework presented in Section **??** can be interpreted as a variational autoencoder[12], conditioned on the stimulus[13], with a (learned) expressive prior[14], and whose latent space is an SDE-induced continuous stochastic process[10]. We can train the model parameters $\Theta = [\theta,\phi]$ by maximizing the evidence lower bound (ELBO) formulated as follows:

$$\mathrm{ELBO}(\Theta;v,y) = \mathbb{E}_Q\left[\sum_{i=0}^{n} \log\, p_\theta(y(t_i) \mid x(t_i), v(t_i))\right] - \mathrm{KL}(Q||P) \tag{6}$$

where the first term represents the log-likelihood of the observations given the latent states and the stimulus, and the second term represents the KL divergence between the prior and approximate posterior.

With the optimization function in place, we can compute the gradients with respect to the model parameters $\Theta$ and backpropagate through our fully differentiable computational graph either via automatic differentiation through the solver operations or via the stochastic adjoint-method[10].

In essence, we can use the variational inference framework to provide an approximate Bayesian treatment of all the inferred values such as the initial state $x_0$, the encoded input $u$, and the UDE parameters $[\theta, \phi]$ ) and update Eq. (6) accordingly. However, this comes at the cost of a more challenging optimization problem to solve. Therefore, pragmatic choices must be made based on the specific application requirements.

## References


1. Oksendal, B. *Stochastic Differential Equations: An Introduction with Applications* (Springer Science & Business Media, 2013).
2. Black, F. & Scholes, M. The pricing of options and corporate liabilities. *J. Polit. Econ.* **81**, 637–654 (1973).
3. Cox, J. C., Ingersoll Jr, J. E. & Ross, S. A. A theory of the term structure of interest rates. In *Theory of Valuation*, 129–164 (World Scientific, 2005).
4. Coffey, W. & Kalmykov, Y. P. *The Langevin Equation: With Applications to Stochastic Problems in Physics, Chemistry and Electrical Engineering*, vol. 27 (World Scientific, 2012).
5. Arató, M. A famous nonlinear stochastic equation (Lotka-Volterra model with diffusion). *Math. Comput. Model.* **38**, 709–726 (2003).
6. Ewens, W. J. *Mathematical Population Genetics: Theoretical Introduction*, vol. 27 (Springer, 2004).
7. Yang, J. & Kushner, H. J. A Monte Carlo method for sensitivity analysis and parametric optimization of nonlinear stochastic systems. *SIAM J. on Control. Optim.* **29**, 1216–1249 (1991).
8. Chow, C. C. & Buice, M. A. Path integral methods for stochastic differential equations. *The J. Math. Neurosci. (JMN)* **5**, 1–35 (2015).
9. Gobet, E. & Munos, R. Sensitivity analysis using Itô–Malliavin calculus and martingales, and application to stochastic optimal control. *SIAM J. on Control. Optim.* **43**, 1676–1713 (2005).
10. Li, X., Wong, T.-K. L., Chen, R. T. & Duvenaud, D. Scalable gradients for stochastic differential equations. In *International Conference on Artificial Intelligence and Statistics*, 3870–3882 (PMLR, 2020).
11. Girsanov, I. V. On transforming a certain class of stochastic processes by absolutely continuous substitution of measures. *Theory Probab. & Its Appl.* **5**, 285–301 (1960).
12. Kingma, D. P. & Welling, M. Auto-encoding variational bayes. *ArXiv Prepr. ArXiv:1312.6114* (2013).
13. Sohn, K., Lee, H. & Yan, X. Learning structured output representation using deep conditional generative models. *Adv. Neural Inf. Process. Syst.* **28** (2015).
14. Ma, T., Chen, J. & Xiao, C. Constrained generation of semantically valid graphs via regularizing variational autoencoders. *Adv. Neural Inf. Process. Syst.* **31** (2018).
